# High-Throughput Sequencing of *Arabidopsis* microRNAs: Evidence for Frequent Birth and Death of *MIRNA* Genes

**DOI:** 10.1371/journal.pone.0000219

**Published:** 2007-02-14

**Authors:** Noah Fahlgren, Miya D. Howell, Kristin D. Kasschau, Elisabeth J. Chapman, Christopher M. Sullivan, Jason S. Cumbie, Scott A. Givan, Theresa F. Law, Sarah R. Grant, Jeffery L. Dangl, James C. Carrington

**Affiliations:** 1 Center for Genome Research and Biocomputing, Department of Botany and Plant Pathology, Oregon State University, Corvallis, Oregon, United States of America; 2 Molecular and Cellular Biology Graduate Program, Oregon State University, Corvallis, Oregon, United States of America; 3 Department of Biology, University of North Carolina, Chapel Hill, North Carolina, United States of America; Michigan State University, United States of America

## Abstract

In plants, microRNAs (miRNAs) comprise one of two classes of small RNAs that function primarily as negative regulators at the posttranscriptional level. Several *MIRNA* genes in the plant kingdom are ancient, with conservation extending between angiosperms and the mosses, whereas many others are more recently evolved. Here, we use deep sequencing and computational methods to identify, profile and analyze non-conserved *MIRNA* genes in *Arabidopsis thaliana*. 48 non-conserved *MIRNA* families, nearly all of which were represented by single genes, were identified. Sequence similarity analyses of miRNA precursor foldback arms revealed evidence for recent evolutionary origin of 16 *MIRNA* loci through inverted duplication events from protein-coding gene sequences. Interestingly, these recently evolved *MIRNA* genes have taken distinct paths. Whereas some non-conserved miRNAs interact with and regulate target transcripts from gene families that donated parental sequences, others have drifted to the point of non-interaction with parental gene family transcripts. Some young *MIRNA* loci clearly originated from one gene family but form miRNAs that target transcripts in another family. We suggest that *MIRNA* genes are undergoing relatively frequent birth and death, with only a subset being stabilized by integration into regulatory networks.

## Introduction

Eukaryotes possess RNA silencing systems to regulate or suppress a range of genes, genetic elements, and viruses [1]. Regulation by RNA silencing can occur at either the transcriptional or posttranscriptional level, although in both cases, silencing is associated with formation of small RNA classes with typical sizes of 21 and 24 nucleotides (nts) [1]–[3]. Small RNA biogenesis occurs from perfect or near-perfect double-stranded RNA (dsRNA) that arises by synthesis of self-complementary foldbacks, by bidirectional transcription, or through the activity of RNA-dependent RNA polymerases (RDR) [1]–[3]. Processing of self-complementary foldback or dsRNA precursors to small RNA duplexes is catalyzed by complexes containing DICER [or DICER-LIKE (DCL)] proteins and dsRNA-binding proteins [1]–[3]. Single-stranded small RNAs then associate with ARGONAUTE (AGO) proteins in effector complexes [4], [5]. For transcriptional silencing, effector complexes associate (directly or indirectly) with factors controlling repressive chromatin, including DNA methylation and histone modification enzymes [6]–[8]. Posttranscriptional silencing effector complexes can mediate irreversible cleavage, translational repression, or subcellular redirection of target transcripts [1]–[3], [9], [10].

The expanse of genetic information regulated posttranscriptionally by small RNAs is potentially large in animals and plants [11]–[13]. In humans, for example, computational and indirect experimental evidence indicates that miRNAs regulate expression of up to 1/3 of all genes [14]–[17]. In plants, far fewer mRNAs are directly regulated by miRNAs, although the direct and indirect consequences of miRNA-directed regulation are significant [11], [12]. This is due to the roles of a large proportion of plant miRNA target transcripts that encode transcription factors required for normal growth, development, hormone response, meristem functions and stress responses [11], [12], [18], [19]. Approximately 21 families of *Arabidopsis* miRNAs and their respective targets are conserved in Rice and/or Poplar [11]. Additionally, there is a growing recognition of significant numbers of miRNAs not conserved in Rice or Poplar, many of which likely arose in the recent evolutionary past [20]–[24]. In some cases, these *MIRNA* loci formed through inverted duplication events that yielded transcripts with self-complementary foldback structure [22]. In fact, the genomes of *Arabidopsis* and other plants contain a wide diversity of non-conserved, local inverted duplications that yield small RNA populations ranging from highly uniform, DCL1-dependent miRNAs (e.g. *MIR163*) to heterogeneous collections of bi-directional short interfering RNAs (siRNAs) formed by multiple DCLs [22], [23], [25], [26].

Deep sequencing methods now provide a rapid way to identify and profile small RNA populations in different plants, mutants, tissues, and at different stages of development. We and others have used high-throughput pyrosequencing to analyze small RNAs across the *Arabidopsis* genome in wild-type and silencing-defective mutants [23], [25], [27]–[29]. In this paper, we identify and analyze non-conserved and recently evolved *Arabidopsis* miRNAs. The data reveal a relatively large number of miRNAs that are so far unique to *Arabidopsis*, and suggest that many miRNAs are spawned and lost frequently during evolution.

## Results and Discussion

### New miRNAs and miRNA target transcripts

Small RNA populations from wild-type (Col-0) plants, from *dcl* (*dcl1-7*, *dcl2-1*, *dcl3-1* and *dcl4-2*) and from *rdr* (*rdr1-1*, *rdr2-1* and *rdr6-15*) mutant plants, as well as from several tissue types of wild-type and *rdr6-15* mutant plants, were sequenced using picoliter-scale pyrosequencing [28], [30]. This yielded quantitative profiling data for several classes of miRNAs and siRNAs [28]. Procedures for sequencing in a multiplexed format, normalization across samples, normalization for multi-locus small RNAs, and viewing at the *Arabidopsis* Small RNA Project database (ASRP) (http://asrp.cgrb.oregonstate.edu/db/) were described [28]. In addition to the populations analyzed previously, small RNAs were profiled from non-infected leaves (15,826 reads), or leaves that were infected for 1 hr (18,368 reads) or 3 hr (10,363 reads) by *Pseudomonas syringae* pv. tomato (DC3000*hrcC*). In total, 218,575 unique small RNAs (663,312 loci), represented by 470,426 reads, were used in this study.

**Figure 1:**
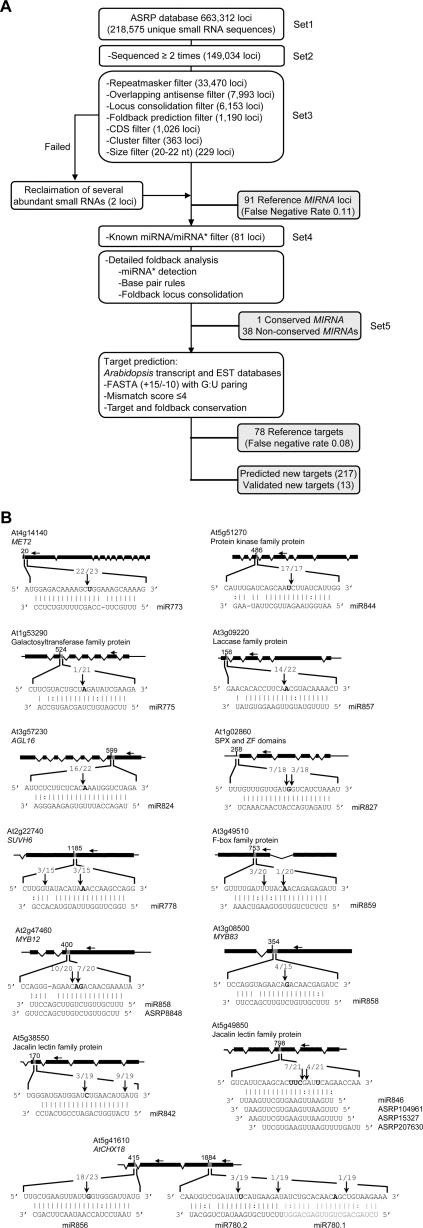
Identification and analysis of *Arabidopsis* miRNAs and targets. (A) Flowchart for the prediction of miRNAs and their targets. (B) Validation of predicted targets for 13 non-conserved miRNAs. Positions of dominant 5′ RACE products (no. 5′ ends at position/total no. 5′ ends sequenced) are indicated by vertical arrows in the expanded regions. Predicted cleavage sites are indicated by a bolded nucleotide at position ten relative to the 5′ end of the miRNA or miRNA-variant. Positions of gene-specific primers are indicated with horizontal arrows above gene diagrams.

A computational analysis to identify new *MIRNA* genes was done using a protocol similar to that of Xie et al. [31] (Figure 1A). All small RNAs from the ASRP database (Set1) were used (Figure 1A). Briefly, all loci from Set1 that yielded at least two reads (Set2) were subjected to Repeatmasker [32] and bidirectional small RNA cluster filters to eliminate siRNAs from repeat sequence classes. Small RNAs that differed at their termini by up to four nucleotide positions were consolidated and passed through a self-complementary foldback screen with settings as described [31]. Small RNAs from coding sequences and complex small RNA clusters, or that were not 20–22 nt in length, were eliminated, yielding 228 loci (Set3). These included 91 of 102 (false negative rate = 0.11) rigorously characterized *MIRNA* genes used as a rule-development and reference set (Table 1) [31]. Failure to identify miR398a, miR399a, miR399d, miR399e, miR399f and miR447c was due to a lack of sequence reads. Known miRNAs also failed due to length (miR163), an incorrectly predicted foldback (miR164b and miR167d) or because the *MIRNA* gene yielded bidirectional small RNAs (miR156d and miR161). Small RNAs from known *MIRNA* genes were then removed from Set3, leaving 79 unique small RNA loci (Figure 1A). Two loci that had each failed at one filter step were reclaimed manually due to their abundance and their dependence on DCL1 (http://asrp.cgrb.oregonstate.edu/db/). The 81 loci (Set4) were then subjected to a detailed foldback analysis where small RNAs from the same foldback were consolidated into a single prospective *MIRNA* locus. A particularly useful, although not absolute, feature was detection of miRNA* sequences, which arise from the opposite foldback arm during DCL1-mediated processing. Detection of miRNA* sequences reveals the functionality of the predicted foldback. miRNA* sequences were detected at relatively low abundance for most reference *MIRNA* families (Table 1).

**Table 1:** Reference set of *Arabidopsis* *MIRNA* families.

*MIRNA* family^a^	Loci	Conserved^b^	miRNA* sequenced	Reads^c^	Target family	Target validation^a^
miR156/miR157	12	Y	Y	15606	Squamosa-promoter binding protein-like (SPL)	Y
miR158	2	N	Y	833	Pentatricopeptide repeat (PPR)	N
miR159/miR319	6	Y	Y	24098	MYB transcription factor	Y
					TCP transcription factor	Y
miR160	3	Y	Y	5783	Auxin response factor (ARF)	Y
miR161	1	N	Y	9191	Pentatricopeptide repeat (PPR)	Y
miR162	2	Y	Y	289	Dicer-like (DCL)	Y
miR163	1	N	Y	263	S-adenosylmethionine-dependent methyltransferase (SAMT)	Y
miR164	3	Y	Y	449	NAC domain transcription factor	Y
miR166/miR165	9	Y	Y	4881	HD-ZIPIII transcription factor	Y
miR167	4	Y	Y	8286	Auxin response factor (ARF)	Y
miR168	2	Y	Y	1286	Argonaute (AGO)	Y
miR169	14	Y	Y	42496	HAP2 transcription factor	Y
miR171/miR170	4	Y	Y	6423	Scarecrow-like transcription factor (SCL)	Y
miR172	5	Y	Y	2720	Apetala2-like transcription factor (AP2)	Y
miR173	1	N	Y	78	TAS1, TAS2	Y
miR390/miR391	3	Y	Y	2889	TAS3	Y
miR393	2	Y	Y	94	Transport inhibitor response 1 (TIR1)/Auxin F-box (AFB)	Y
					bHLH transcription factor	Y
miR394	2	Y	Y	47	F-box	Y
miR395	6	Y	Y	14	ATP-sulfurylase (APS)	Y
					Sulfate transporter (AST)	Y
miR396	2	Y	Y	408	Growth regulating factor (GRF)	Y
miR397	2	Y	N	290	Laccase (LAC)	Y
miR398	3	Y	Y	42	Copper superoxide dismutase (CSD)	Y
					Cytochrome-c oxidase	Y
miR399	6	Y	Y	50	E2 ubiquiting-conjugating protein (E2-UBC)	Y
miR400	1	N	Y	18	Pentatricopeptide repeat (PPR)	N
miR402	1	N	N	10	HhH-GPD base excision DNA repair	N
miR403	1	Y	Y	29	Argonaute (AGO)	Y
miR408	1	Y	Y	42	Laccase (LAC)	Y
					Plantacyanin-like (PCL)	N
miR447	3	N	N	25	2-phosphoglycerate kinase-related (2-PGK)	Y

a Reviewed in Jones-Rhoades et al. [11].

b Conserved between *A. thaliana* and *P. trichocarpa*.

c Number of reads are from all libraries in the ASRP database (http://asrp.cgrb.oregonstate.edu/db).

Reads for each locus encompass the defined miRNA sequence ±4 nts on each side.

Thirty-nine loci (Set5) beyond the reference set emerged as prospective miRNAs, including 25 for which miRNA* sequences were detected (Table 2). Eight miRNAs from Set5 were detected recently as miRNAs by Lu et al. [23]. Only one sequence, corresponding to *Populus trichocarpa* miR472 [24], was conserved in Poplar based on sequence/foldback similarity and target conservation. None of the new sequences were conserved in Rice. In several cases (miR830, miR833, miR851, miR861, miR862, miR863, miR864, miR865 and miR866), due to the ratio of small RNA sequences from both the 5p and 3p strands of the foldback, it was not clear which small RNA should be designated as the miRNA. In such cases, both the 5p and 3p strand sequences are designated (Table 2). In total, at least 70 *MIRNA* families were detected in at least one sequenced population (Tables 1 and 2, and references therein). Most miRNAs were lost or underrepresented in the *dcl1-7* mutant (http://asrp.cgrb.oregonstate.edu/db/).

Target RNAs were predicted from both transcript and EST databases for the 39 miRNAs in Set5 by the method of Allen et al. [22], which is based on additive, position-dependent mispair penalties. Seventy-eight or 69 of 85 validated, reference miRNA targets were predicted with a threshold score of 4 or 3.5, respectively (false negative rate = 0.08 or 0.19; Figure 1A). A total of 142 targets were predicted for the Set5 miRNAs, using the more conservative 3.5 score threshold (Table 2). These included targets predicted for both 5p and 3p strands for eight *MIRNA* loci. The 3.5 score threshold was used to maintain specificity, although at the cost of sensitivity, in the target prediction algorithm.

**Table 2:** New or recently identified miRNAs

					Reads^b^			
MIRNA family	Sequence	Loci	Conserved^a^	miRNA* sequenced	miRNA	miRNA*	Validated or predicted target family^c^	Validated or top predicted targets^c^
***MIRNA*** **s with validated targets**								
miR472	UUUUUCCUACUCCGCCCAUACC	1	Y	Y	9 (206)	2 (51)	**CC-NBS-LRR**	**At1g51480 (1)** d, **At5g43740 (1)** d, At1g12290 (1.5), [8]
miR773	UUUGCUUCCAGCUUUUGUCUCC	1	N	N	11 (735)	0 (0)	**DNA (cytosine-5-)-methyltransferase**	**At4g14140 (2)**, At4g08990 (3)
miR774	UUGGUUACCCAUAUGGCCAUC	1	N	N	0 (348)	0 (0)	**F-box**	**At3g19890 (1)** d, At3g17490 (2.5), At3g17265 (3.5)
miR775	UUCGAUGUCUAGCAGUGCCA	1	N	Y	1362 (1532)	5 (3)	**Galactosyltransferase**	**At1g53290 (2.5)**
miR778	UGGCUUGGUUUAUGUACACCG	1	N	Y	0 (2)	1 (7)	**SET-domain**	**At2g22740 (1.5)**, At2g35160 (3.5)^e^
miR780.1	UCUAGCAGCUGUUGAGCAGGU	1	N	Y	57 (266)	0 (118)	**Cation/hydrogen exchanger**	**At5g41610 (3.5)**, At4g33260 (3.5)
miR780.2	UUCUUCGUGAAUAUCUGGCAU							
miR824	UAGACCAUUUGUGAGAAGGGA	1	N	Y	261 (2646)	399 (2)	**MADS-box transcription factor**	**At3g57230 (0.5)**
miR827	UUAGAUGACCAUCAACAAACU	1	N	Y	11 (20)	0 (1)	**SPX (SYG1/Pho81/XPR1) domain/Zinc finger (C3HC4-type)**	**At1g02860 (1)**
miR842	UCAUGGUCAGAUCCGUCAUCC	1	N	Y	2 (97)	2 (0)	**Jacalin lectin**	**At5g38550 (2.5)**, At1g60130 (2.5), At1g52120 (2.5), [3]
miR844	AAUGGUAAGAUUGCUUAUAAG	1	N	Y	58 (1)	2 (0)	**Kinase**	**At5g51270 (3.5)**
miR846	UUGAAUUGAAGUGCUUGAAUU	1	N	N	72 (0)	0 (0)	**Jacalin lectin**	**At5g49850 (2.5)**, At5g49870 (2.5)^e^, At2g25980 (2.5)^e^, [8]
miR856	UAAUCCUACCAAUAACUUCAGC	1	N	Y	62 (7)	9 (0)	**Cation/hydrogen exchanger, Zinc transporter**	**At5g41610 (1)**, At2g46800 (2.5)^e^
miR857	UUUUGUAUGUUGAAGGUGUAU	1	N	N	59 (0)	0 (0)	**Laccase**	**At3g09220 (2)**
miR858	UUUCGUUGUCUGUUCGACCUU	1	N	N	55 (4)	0 (0)	**MYB transcription factor**	**At2g47460 (2.5)**, **At3g08500 (3)**, At5g35550 (3), [6]
miR859	UCUCUCUGUUGUGAAGUCAAA	1	N	N	2 (5)	0 (0)	**F-box**	At3g17265 (0.5)^e^, At5g36200 (1)^e^, **At3g49510 (1.5)**, [32]
***MIRNA*** **s with only predicted targets, or no predicted targets**								
miR771	UGAGCCUCUGUGGUAGCCCUCA	1	N	Y	16 (906)	0 (44)	-	-
miR776	UCUAAGUCUUCUAUUGAUGUUC	1	N	N	1439 (487)	0 (0)	Serine/threonine kinase	At5g62310 (3)^e^
miR777	UACGCAUUGAGUUUCGUUGCUU	1	N	N	8 (80)	0 (0)	-	-
miR779	UUCUGCUAUGUUGCUGCUCAUU	1	N	N	2 (98)	0 (0)	-	-
miR781	UUAGAGUUUUCUGGAUACUUA	1	N	Y	0 (77)	1 (0)	CD2-binding, MCM	At5g23480 (2.5)^e^, At1g44900 (3)
miR823	UGGGUGGUGAUCAUAUAAGAU	1	N	N	107 (1)	0 (0)	Chromomethylase	At1g69770 (2.5)^e^
miR825	UUCUCAAGAAGGUGCAUGAAC	1	N	N	120 (0)	0 (0)	Remorin, zinc finger homeobox family, frataxin-related	At2g41870 (2.5)^e^, At5g65410 (3)^e^, At4g03240 (3)^e^, [1]
miR829.2	AGCUCUGAUACCAAAUGAUGGAAU	1	N	Y	134 (41)	3 (25)	-	-
miR830-5p	UCUUCUCCAAAUAGUUUAGGUU	1	N	Y	2 (1)	-	RanBP1 domain, kinesin motor-related	At1g52380 (3)^e^, At3g45850 (3.5)^e^
miR830-3p	UAACUAUUUUGAGAAGAAGUG				-	3 (21)	-	-
miR833-3p	UAGACCGAUGUCAACAAACAAG	1	N	Y	5 (2)	-	-	-
miR833-5p	UGUUUGUUGUACUCGGUCUAG				-	2 (1)	F-box	At1g77650 (3.5)^e^
miR840	ACACUGAAGGACCUAAACUAAC	1	N	Y	20 (1)	7 (116)	WHIRLY transcription factor	At2g02740 (0)^e^
miR843	UUUAGGUCGAGCUUCAUUGGA	1	N	Y	7 (0)	2 (0)	F-box, 1-aminocyclopropane-1-carboxylate synthase	At3g13830 (0.5)^e^, At1g11810 (2.5)^e^, At2g22810 (3)
miR845a	CGGCUCUGAUACCAAUUGAUG	2	N	Y	670 (21)	1 (40)	-	-
miR845b	UCGCUCUGAUACCAAAUUGAUG							
miR851-5p	UCUCGGUUCGCGAUCCACAAG	1	N	Y	3 (281)	1 (1)	-	-
miR852	AAGAUAAGCGCCUUAGUUCUGA	1	N	Y	3 (84)	0 (1)	ATPase	At5g62670 (3)^e^
miR853	UCCCCUCUUUAGCUUGGAGAAG	1	N	N	2 (0)	0 (0)	-	-
miR860	UCAAUAGAUUGGACUAUGUAU	1	N	Y	14 (15)	0 (1)	Histone deacetylase, ferrochelatase, RNA recognition motif	At5g26040 (0)^e^, At5g26030 (0.5)^e^, At3g12640 (3.5)
miR861-3p	GAUGGAUAUGUCUUCAAGGAC	1	N	Y	6 (2)	-	-	-
miR861-5p	CCUUGGAGAAAUAUGCGUCAA				-	1 (8)	-	-
miR862-5p	UCCAAUAGGUCGAGCAUGUGC	1	N	Y	5 (0)	-	-	-
miR862-3p	AUAUGCUGGAUCUACUUGAAG				-	2 (0)	-	-
miR863-3p	UUGAGAGCAACAAGACAUAAU	1	N	Y	5 (0)	-	-	-
miR863-5p	UUAUGUCUUGUUGAUCUCAAU				-	2 (0)	Kinase, Legumain (C13 protease)	At2g26700 (3), At1g62710 (3.5)^e^
miR864-5p	UCAGGUAUGAUUGACUUCAAA	1	N	Y	3 (0)	-	Triacylglycerol lipase	At1g06250 (3)^e^
miR864-3p	UAAAGUCAAUAAUACCUUGAAG				-	2 (0)	Expressed protein	At4g25210 (3)^e^
miR865-5p	AUGAAUUUGGAUCUAAUUGAG	1	N	Y	3 (0)	-	Serine carboxypeptidase, sulfate transporter	At5g42240 (3.5)^e^, At3g51895 (3.5)^e^
miR865-3p	UUUUUCCUCAAAUUUAUCCAA				-	1 (0)	DEAD box RNA helicase, DNA-binding bromodomain-containing protein	At2g07750 (3)^e^, At2g34900 (3)^e^, At1g03770 (3.5), [6]
miR866-3p	ACAAAAUCCGUCUUUGAAGA	1	N	Y	2 (0)	-	Kinase, electron transport SCO1/SenC, NAD-dependent G-3-P dehydrogenase	At4g21400 (3)^e^, At4g39740 (3), At2g41540 (3)^e^, [4]
miR866-5p	UCAAGGAACGGAUUUUGUUAA^f^				-	0 (5)	Expressed protein, C2 domain-containing protein	At4g21700 (3)^e^, At1g66360 (3.5)^e^
miR867	UUGAACAUGGUUUAUUAGGAA	1	N	N	30 (0)	0 (0)	PHD finger-related/SET domain, kinase, phospholipase/carboxylesterase	At4g27910 (3.5)^e^, At3g17750 (3.5)^e^, At3g15650 (3.5)^e^
miR868	CUUCUUAAGUGCUGAUAAUGC	1	N	N	9 (1)	0 (0)	-	-
miR869.1	UCUGGUGUUGAGAUAGUUGAC	1	N	N	11 (5)	0 (0)	-	-
miR869.2	AUUGGUUCAAUUCUGGUGUUG							
miR870	UAAUUUGGUGUUUCUUCGAUC	1	N	N	4 (32)	0 (0)	-	-

a Conserved between *A. thaliana* and *P. trichocarpa*.

b Number of reads are from all libraries in the ASRP (http://asrp.cgrb.oregonstate.edu/db) and MPSS Plus (http://mpss.udel.edu/at/) databases. Reads for each locus encompass the defined miRNA/miRNA* sequence ±4 nts on each side. ASRP (MPSS Plus).

c Top three predicted targets with a score of 3.5 or less are listed with their score in parentheses. Targets validated by 5′ RACE are in bold. Remaining number of targets predicted with a score of 3.5 or less are listed in square brackets (Table S1). Dashes indicate no predicted targets with a score of 3.5 or less.

d Targets validated by Lu et al. [23].

e Target tested but failed in 5′RACE validation assays.

f Seventeen nt MPSS Plus signature was extended 4 nts on the 3′ end.

Top-scoring predicted targets for most miRNAs from Set5, and two that were not found in this study (miR778 and miR781) [23], were tested using a standard 5′RACE analysis to detect cleavage events at predicted sites opposite nucleotide 10 from the 5′ end of the small RNA. The 5′RACE assays were done using two gene-specific primer sets with RNA from whole seedling and inflorescence tissues. Thirteen targets for 13 miRNAs were validated, although evidence supporting miR775- and miR859-guided cleavage (At1g53290 and At3g49510, respectively) was weak (Figure 1B, Table 2). Evidence for miR846- and miR844-guided cleavage should also be interpreted cautiously, as targets were not cleaved at the canonical position. These were considered validated because both miRNAs had multiple sequenced variants that had predicted target sites encompassing the observed cleavage sites (Figure 1B; http://asrp.cgrb.oregonstate.edu/db/). Additionally, targets for miR472 and miR774 were validated recently [23]. Each target-validated miRNA was represented by at least ten reads or had a predicted miRNA* sequence represented with at least one read in the database, except for miR774 and miR778 which were not sequenced here (Table 2). Twenty-two predicted miRNAs that were sequenced at least two times, or that had miRNA* sequences represented in the ASRP or MPSS databases [33], failed at the target validation step (Table 2).

The relatively high proportion of target validation failures could be due to erroneous target predictions, low-abundance targets or miRNA-guided cleavage products, or low-abundance miRNAs with limited or no activity. Additionally, for 13 miRNAs, no targets were predicted at a score threshold of 3.5 (Table 2). While it is possible that many of these function to guide cleavage of unpredicted target RNAs, it is also possible that miRNAs exist without actual targets (see below).

**Figure 2:**
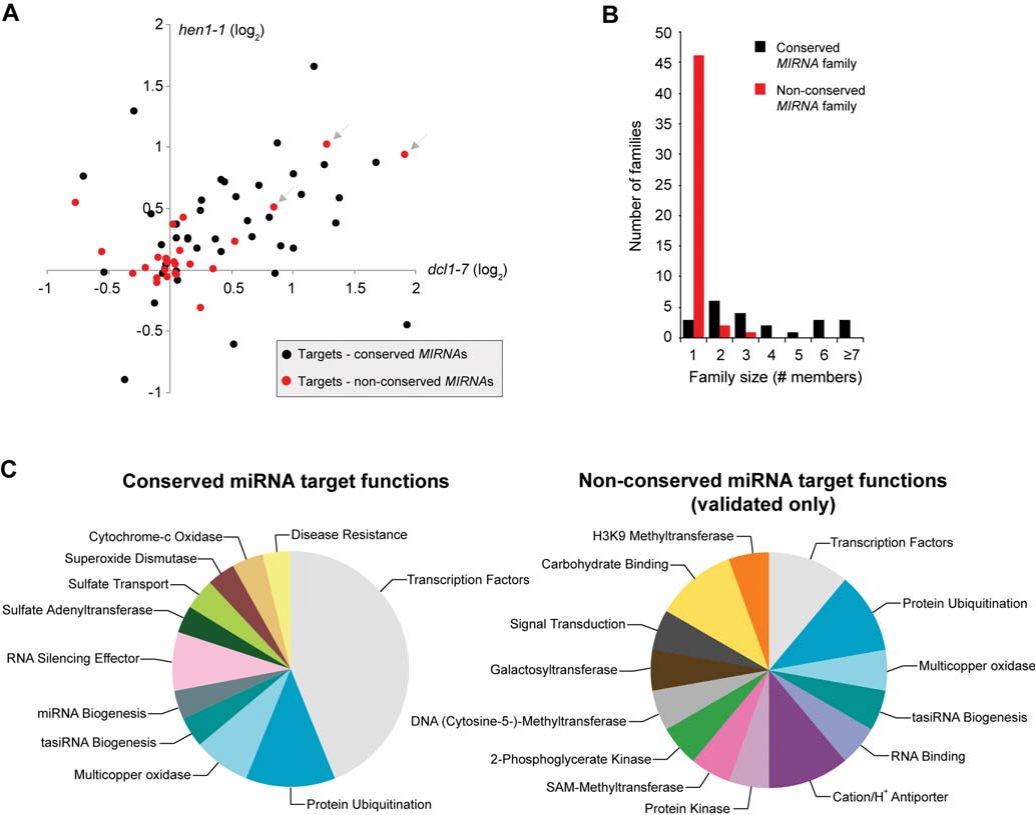
Comparison of conserved and non-conserved *MIRNA* families. (A) Effect of *dcl1-7* and *hen1-1* mutations on levels of target transcripts for conserved (black) and non-conserved (red) miRNAs. Expression data are shown for two validated or high-confidence predicted targets, if available, for each family. Arrows indicate targets for miR824 (*AGL16*), miR858 (*MYB12*) and miR161.1 (At1g63130, a *PPR* gene). (B) Numbers of gene family members for conserved and non-conserved *MIRNA*s (Tables 1 and 2). (C) Relative numbers of miRNA target family functions for conserved and non-conserved miRNAs (Tables 1 and 2). Only target classes that have been validated experimentally are included. Note that Table 2 shows many *MIRNA* families with weak or no predicted targets, and these are not represented in the chart.

Several new target families are worth noting. First, at least two targets are clearly under negative regulation by miRNAs in inflorescence tissue. Transcripts from *AGL16* (At3g57230, MADS-box) and *MYB12* (At2g47460, R2R3-MYB family) genes are targeted by miR824 and miR858, respectively, and are each up-regulated by 2–4 fold in *dcl1-7* and *hen1-1* mutants (Table 2, Figure 2A). Functions for these specific transcription factors are not known. Second, the transcript for the cation/hydrogen antiporter gene *CHX18* is targeted by both miR780 and miR856 (Table 2, Figure 1B). Dual targeting of the *CHX18* (At5g41610) transcript may lead to secondary, 21 nt siRNAs that arise through the RDR6/DCL4-dependent pathway ([29], [34]–[39]; data not shown). miR856 may also target an unrelated transcript encoding the efflux protein ZINC TRANSPORTER OF ARABIDOPSIS THALIANA1 (ZAT1). Third, miR857 was validated to target the *LAC7* (At3g09220) transcript, which encodes a laccase family protein with predicted multicopper oxidase function. This represents the third miRNA family to target the laccase gene family [18], [40], [41]. In fact, at least seven gene or domain families (encoding MYB, PENTATRICOPEPTIDE REPEAT (PPR), AUXIN RESPONSE FACTOR (ARF), AGO, F-box domain, kinase and LAC proteins) are now known to be targeted by multiple miRNA families [11].

### Direct sequencing as a miRNA expression profiling tool

**Figure 3:**
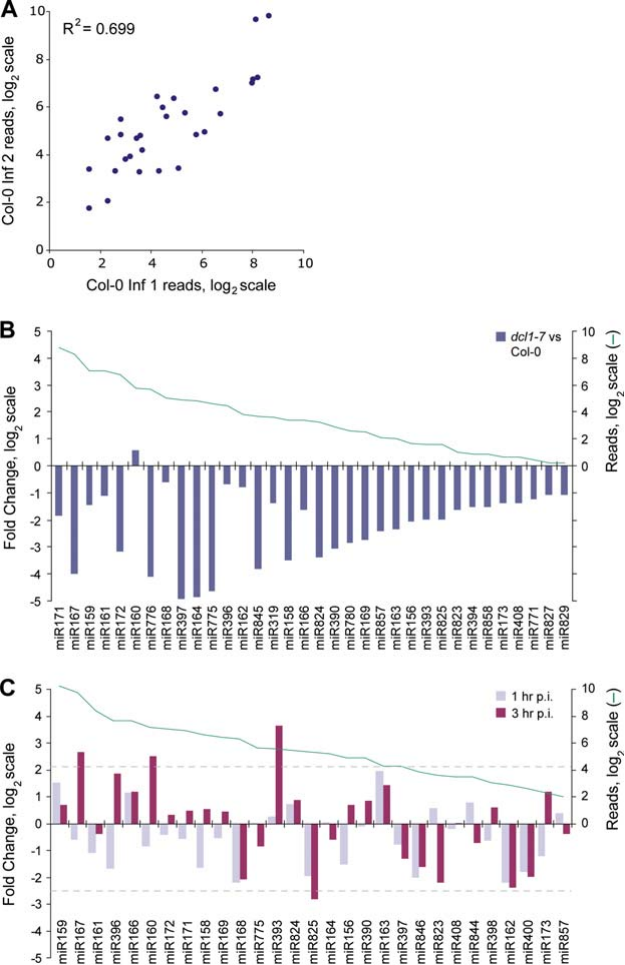
Expression profiling of *MIRNA* families using high-throughput pyrosequencing. (A) Comparison of most-abundant miRNA families between biological replicates of Col-0 inflorescence (inf.) tissue. Normalized reads for each miRNA family member were consolidated. Note that *MIR159* and *MIR319* derived members were counted separately, even though they are frequently assigned to the same family [31], [57]. (B) Fold-change of miRNAs in *dcl1-7* inflorescence versus Col-0 inflorescence (left axis, bars). Total number of reads for each family is indicated (right axis, green line). As the *dcl1-7* mutant contained no reads for many miRNA families, fold-change was calculated using normalized reads+1. This had the effect of dampening fold-change values for low-abundance families. (C) Fold-change of miRNA family reads in leaves at 1 hr and 3 hr post-inoculation with *P. syringae* (DC3000*hrcC*) (left axis, bars). Fold-change relative to uninoculated leaves was calculated based on normalized reads as described in panel (B). Total number of reads in the control and inoculated samples is shown (right axis, green line). Grey dashed lines indicate the *p* = 0.05 upper and lower thresholds.

The primary purpose of this study was to analyze new *MIRNA* loci. Nevertheless, the data from multiple mutants and treatments allowed us to explore the suitability of direct sequencing as a miRNA profiling tool. This was done in several ways. First, the reproducibility of the pyrosequencing method was analyzed using two normalized biological replicates [Col-0 inf. 1 (35,666 reads) and Col-0 inf. 2 (42,917 reads)]. miRNA families represented in both samples by at least three reads were compared and yielded an 83% correlation (Figure 3A). This experimental approach is essentially a random sampling from a very large population of small RNAs and hence we anticipate that this correlation would increase with greater sequencing depth.

We next compared normalized expression profiles of miRNA families in Col-0 (inflorescence tissues) and *dcl1-7* (inflorescence tissues) mutant plants. As expected, based on the requirement for DCL1 in miRNA precursor processing, levels of nearly all miRNA families were decreased in *dcl1-7* mutant plants (Figure 3B). A few miRNAs were largely unaffected in *dcl1-7* plants. This likely reflects the known residual activity in, and the insensitivity of some miRNAs to, the partial-loss-of-function *dcl1-7* mutant allele [22].

To extend our small RNA profiling beyond different tissues and developmental stages, we generated expression profiles of miRNA families in leaves following infection by *P. syringae* pv. tomato (DC3000*hrcC*) at 0, 1 and 3 hr post-inoculation (p.i.) timepoints. This bacterium is unable to cause disease, because it is mutated in the key virulence factor delivery machine, the type III secretion system. This strain does trigger a robust basal defense response in *Arabidopsis* [42]. As predicted [43], miR393 was strongly up-regulated (10-fold at 3 hr p.i.) (Figure 3C). miR393 targets mRNAs encoding the auxin receptor, TRANSPORT INHIBITOR RESPONSE1 (TIR1), and related proteins [18], [43]. Two additional miRNA families, miR160 and miR167, were significantly elevated at 3 hr p.i. by 5-fold and 6-fold, respectively (Figure 3C). miR160 and miR167 each target mRNAs encoding members of the ARF family of transcription factors [19]. These data suggest that basal defense responses triggered by *P. syringae* pv. tomato (DC3000*hrcC*) include miRNA-mediated suppression of multiple components of auxin signaling pathways. Hence, our data extend the conclusions of Navarro et al. [43]. miR825, for which several targets were predicted but not validated (Table 2), was significantly down-regulated 3 hr p.i. (Figure 3C).

Thus, direct sequence-based profiling shows considerable promise in revealing dynamic changes in miRNA populations, although shallow sequencing depth will limit applicability of the method. While both open-ended platforms (such as direct sequencing) and closed-ended platforms (such as solid-state microarrays) can be used to profile known miRNAs, the sequence-based profiling approach affords discovery of previously unknown miRNAs. It also allows discrete measurements of complex mixtures of small RNAs that arise in heterogeneous populations from loci with diffuse boundaries [23], [27], [28].

### Conserved vs. non-conserved miRNAs

Twenty-two and 20 *MIRNA* families (Tables 1 and 2) are conserved in Poplar and Rice, respectively. Conserved families in *Arabidopsis* were designated by having identical or related (three or fewer nucleotide substitutions) sequences, and at least one conserved target transcript, in either Poplar or Rice. A series of general and functional comparisons between conserved and non-conserved families was done. Whereas 19 of 22 conserved *Arabidopsis* miRNA families were represented at multiple loci, only three (*MIR158*, *MIR447* and *MIR845*) of 48 non-conserved miRNAs were members of multigene families (Figure 2B). Expansion of multigene *MIRNA* families has occurred through tandem and segmental duplications, as well as polyploidization events [22], [44], [45]. The preponderance of single gene families among the non-conserved miRNAs is consistent with recent evolutionary derivation.

Functions of conserved miRNA target genes, as a group, are less diverse than functions for non-conserved miRNA target genes (only validated or high-confidence targets were compared). This is due primarily to the high proportion of target mRNAs encoding transcription factors for conserved families (Table 1 and 2, Figure 2C). As pointed out clearly before [18], [19], the vast majority of transcription factor families targeted by conserved miRNAs participate in developmental pathways, including those specifying meristem functions, organ polarity, cell division control, organ separation and hormone responses. Non-conserved miRNA target genes encode a broad range of proteins, including a limited number of transcription factors (Table 2, Figure 2C). Interestingly, several non-conserved miRNAs (miR161 and miR400) target transcripts from a clade within the large *PPR* family [19], [41]. Another non-conserved miRNA, miR173, targets tasiRNA primary transcripts (*TAS1* and *TAS2*) [34], which in turn yield siRNAs that also target several of the miR161- and miR400-targeted *PPR* transcripts ([29]; data not shown).

To what extent do the non-conserved miRNAs negatively regulate target genes? The sensitivity of target genes of conserved and non-conserved miRNAs was analyzed by transcript profiling in wild-type (Col-0 and La-*er*) plants and mutant plants with general miRNA deficiencies (*dcl1-7* and *hen1-1*). To avoid biasing the analysis with miRNAs that target disproportionately high numbers of target mRNAs, such as miR161 and *PPR* target gene family members, the number of genes analyzed for each miRNA family was limited to two validated targets, or two predicted targets with the lowest scores. For miRNAs that have been shown to target multiple gene families, such as miR395, two targets from both gene families were analyzed. A scatterplot of fold-change in *dcl1-7* and *hen1-1* mutants for each target was generated. As shown previously [34], conserved miRNA target transcripts displayed a generally elevated pattern in both mutants (Figure 2A). In contrast, most target transcripts of non-conserved miRNAs were clustered around the origin, indicating that most were insensitive to either the *dcl1-7* or *hen1-1* mutation (Figure 2A). The exceptions that were affected at levels of 1.6-fold or greater in either mutant included transcripts from *AGL16*, *MYB12* and a *PPR* gene (At1g63130), which were targeted by miR824, miR858 and miR161.1, respectively (Figure 2A). While some of these data may be skewed by tissue-specific expression patterns of miRNAs and target genes, the general trend for non-conserved miRNAs having fewer effects on target transcript levels is clear. We suggest that, in contrast to the vast majority of conserved miRNAs, a high proportion of the non-conserved miRNAs are not integrated as dominant factors within regulatory networks.

### Recent evolution of non-conserved *MIRNA* loci

Previously, we identified a number of small RNA-generating loci with the potential to yield transcripts with self-complementary foldback potential and with extensive similarity to protein-coding gene family sequences [22]. Whereas the majority of these loci yield complex populations of siRNAs, two (*MIR161* and *MIR163*) yield functional, non-conserved miRNAs [22]. This led to the idea that aberrant replication/recombination or transposition events from expressed gene sequences can spawn new small RNA-generating loci with the potential to evolve into *MIRNA* genes that regulate members of the originating family.

**Figure 4:**
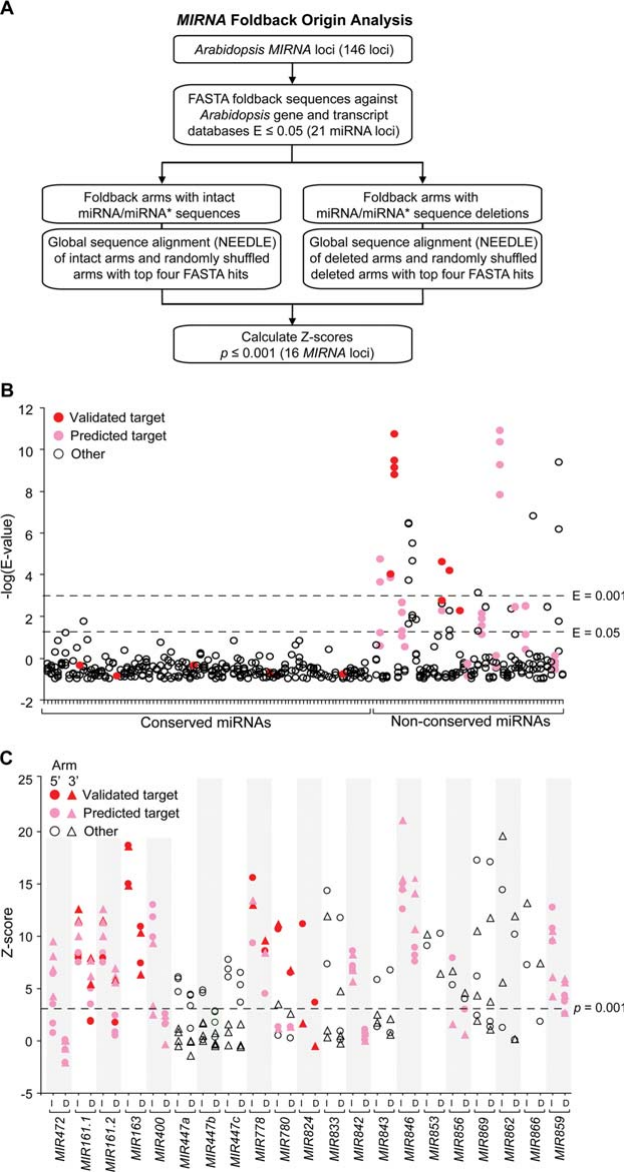
Identification of *MIRNA* foldbacks with similarity to protein-coding genes. (A) Flowchart for identification of *MIRNA* foldbacks with similarity, extending beyond the miRNA target site, to protein-coding genes. (B) *Arabidopsis* gene or transcript hits in FASTA searches using foldback sequences for all conserved and non-conserved *MIRNA* loci (Tables 1 and 2). The top four hits based on E-values are shown. (C) Z-scores for the Needleman-Wunche alignment values from *MIRNA* foldback arms with top four gene or transcript FASTA hits. Alignments were done with intact foldback arms (I), and with foldback arms in which miRNA or miRNA-complementary sequences were deleted (D). Z-scores were derived from standard deviation values for alignments of randomized sequences. In (B) and (C), a red symbol represents an experimentally validated target, a pink symbol indicates a gene from a validated target family, and an open symbol indicates a gene that is distinct from either the validated or predicted target family.

This idea was tested more rigorously with the expanded set of *MIRNA* loci. Foldback sequences for each *MIRNA* locus (Tables 1 and 2) were used in FASTA searches against *Arabidopsis* transcript and gene databases (Figure 4A). Nearly all foldback sequences from conserved miRNAs had hits with non-significant E-values greater than 0.05 (Figure 4B). In contrast, 19 of 48 non-conserved miRNA foldback sequences had at least one hit with an E-value lower than 0.05 (Figure 4B). Similarity or complementarity was detected on both 5′ and 3′ arms containing miRNA or miRNA* sequences.

**Figure 5:**
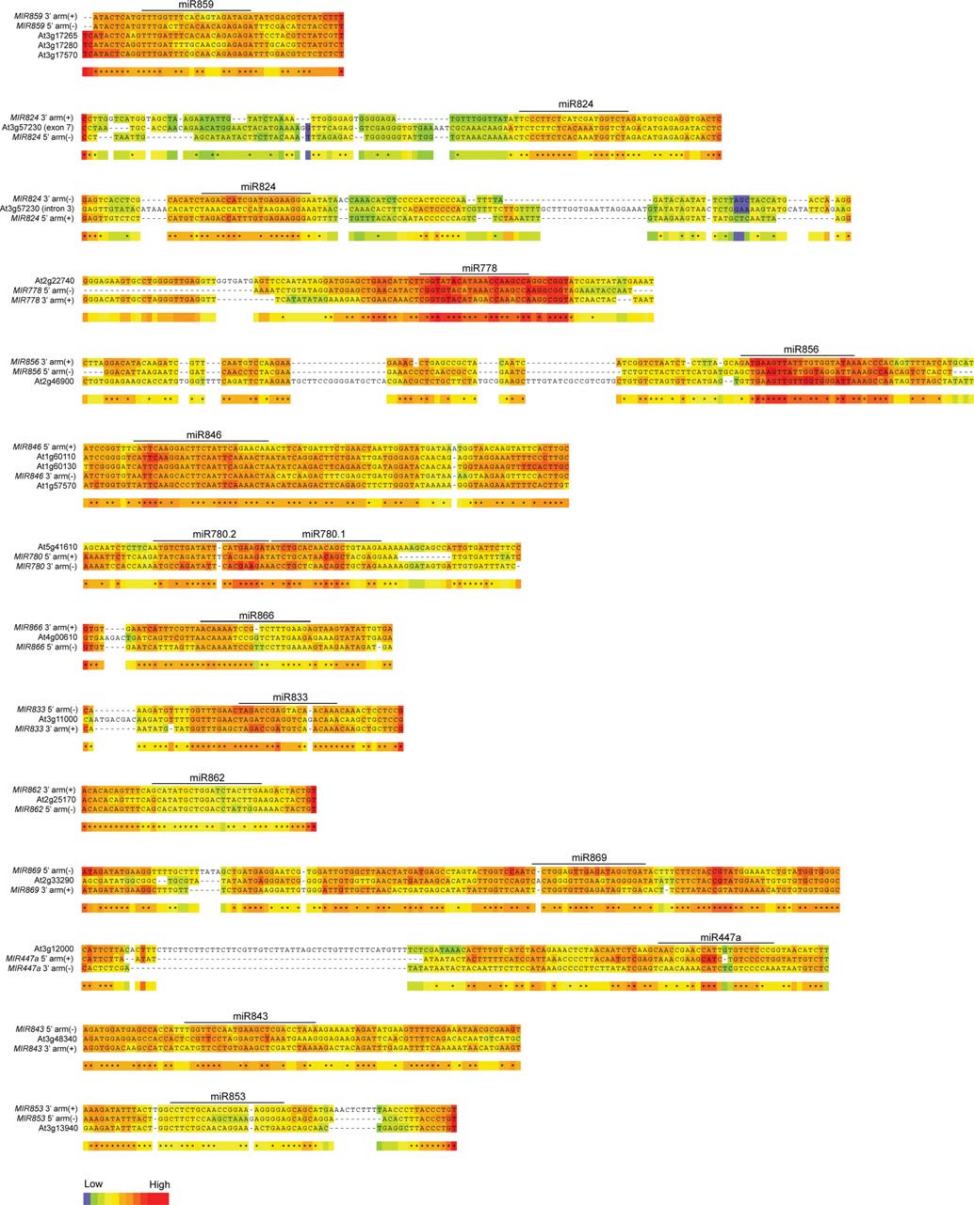
Similarity between *MIRNA* foldback arms and protein-coding genes. Each alignment contains the coding strand for 1–3 genes, the miRNA* arm, and the miRNA arm. Orientation of the foldback arms is indicated by (+) for authentic polarity and (−) for the reverse complement polarity. Two alignments are given for *MIR824* because the two arms are each most similar to distinct, duplicated regions within the *AGL16* gene (At3g57230). Alignments were generated using T-COFFEE. Colors indicate alignment quality in a regional context.

It is important to recognize that *MIRNA* loci contain two regions – the miRNA and miRNA-complementary region (largely overlapping with the miRNA*) – with relatively high levels of complementarity or similarity to target genes. To eliminate the potential misleading influence of these sequences, which may be under selection due to the requirement for complementarity between miRNAs and their targets, on the similarity test, each foldback arm with hits (E<0.05) in the FASTA search was analyzed independently with and without the miRNA or miRNA-complementary sequences. The top four FASTA hits (Figure 4A) were aligned with the intact or deleted arms using a global sequence alignment method (NEEDLE). *MIR163* and *MIR161* were analyzed in detail previously [22] and were included here as controls. Two foldback arm sets from *MIR161*, with miR161.1/miR161.1-complementary and the overlapping miR161.2/miR161.2-complementary sequences deleted independently, were analyzed. Sixteen foldbacks, including the *MIR161* and *MIR163* controls, contained at least one arm with similarity or complementarity (NEEDLE score with p<0.001) to one or more genes when an alignment was done with both intact and deleted arms (Figure 4C, Table S3). For all hits with the deleted arms, the top-score gene alignments identified sequences that were immediately flanking the region with similarity or complementarity to miRNA regions (Figure 5). Further, in all cases in which multiple genes were hit with intact and deleted arms, the genes were closely related members of a single family. Thus, over 30% of the non-conserved *MIRNA* loci show evidence of common origin with specific genes or gene families.

Interestingly, the two *MIR824* arms aligned best with distinct regions within one gene (*AGL16*). The miR824 arm was complementary to a region from exon VIII, and the miR824* arm was most similar to a duplicated region located within intron III. The two arms from *MIR846* were most similar to two adjacent but distinct regions of jacalin domain-containing genes or pseudogenes (At1g57570 and At1g61230). It is likely, therefore, that some recently evolved *MIRNA* loci arose through juxtapositioning of sequences from two related duplicated sequences.

The protein-coding genes with extended *MIRNA* foldback arm similarity were analyzed in more detail. The intact foldback arms from 13 of the 16 gene-similar *MIRNA* loci were aligned with up to three gene sequences, and alignment quality was displayed using heat maps (Figure 5; *MIR161*, *MIR163* and *MIR447c* were not included). This revealed several sequence conservation patterns. For several *MIRNA* arm-gene alignments, including those containing miR778, miR780, miR824 and miR856 sequences, the aligned region containing miRNA sequences was clearly more conserved than the remaining arm segments (Figure 5). This suggests that selection may be operating on several of the recently evolved miRNA sequences, and is indirect evidence for functional significance of the miRNA. Indeed, miR824 targets the *AGL16* transcript, which is under miRNA pathway-dependent repression (Figure 2A).

**Figure 6:**
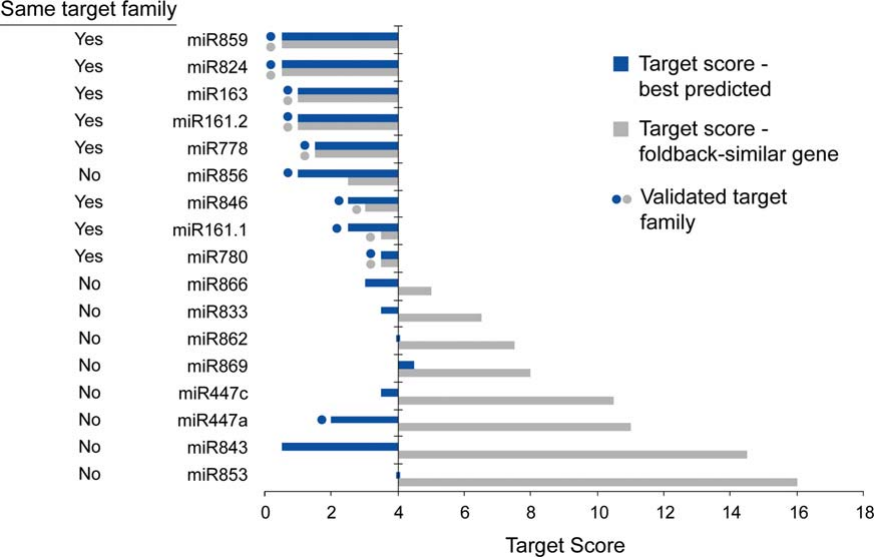
Targeting specificity of recently evolved *MIRNA*s. Two target prediction scores are shown for each of 16 miRNAs: best overall predicted target score (blue) and target scores calculated for *MIRNA* foldback-similar genes (grey). Left column indicates whether or not the best overall predicted target gene is in the same family as the foldback-similar gene. A dot indicates that the predicted gene is in an experimentally validated target family. Two calculations corresponding to the two major populations from the *MIR161* locus (miR161.1 and miR161.2) are shown. The identities of targets are listed in Supplemental Table S3. The plot is centered on a target prediction score of 4, as this corresponds to the upper limit of a reasonable prediction.

For several other *MIRNA* arm-gene alignments, such as those containing miR862, miR447a and miR853, the miRNA-region of the alignment is similar to, or weaker than, the alignment containing flanking arm sequences (Figure 5). In fact, among the 16 *MIRNA*-similar gene sets identified in this analysis (Table S3), only seven represented sets that corresponded to the best predicted or validated miRNA-target pair (Figures 4 and 6). In other words, nine of the miRNAs from loci with similarity to protein-coding genes were predicted to target transcripts from different genes (based on best target prediction scores). The target scores for eight of these sets were clearly weak or functionally implausible (Figure 6, Table S3). Interestingly, miR447a and miR856 were validated to target transcripts in gene families distinct from those similar to the foldback sequences (Figure 1B, Table 2; [34]). The miR447 family appears to have acquired novel target specificity and lost sequence-of-origin specificity, while miR856 may have acquired dual-targeting specificity (Figure 6, Table 2, Table S3).

The picture that emerges from analysis of the non-conserved *Arabidopsis* miRNAs appears to show that new *MIRNA* loci are forming frequently through duplication events. These newly evolved *MIRNA* loci may first pass through a stage in which heterogeneous populations of siRNA-like sequences are generated, especially if the duplicated locus and resulting foldback sequence is large [22]. This, of course, assumes that the newly spawned sequence is proximal to a functional promoter. Computational evidence indicates that formation of *MIR163* through inverted duplication of SAMT methyltransferase-like sequences also involved duplication of the gene-of-origin promoter [46]. Given that DCL1 has limited or insignificant activity on perfectly-paired dsRNA, acquisition of DCL1-dependence and subsequent formation of discrete small RNA products from the foldback precursor may require accumulation of drift mutations that result in foldback mispairs. From this point, we envision three evolutionary fates. The first and perhaps most common is continued sequence drift through mutation, decay of both targeting capacity and promoter (if not required for other functions) sequences, and eventual death of the locus. New *MIRNA* genes with neutral effects are predicted to take this route. The second fate is stabilization of the miRNA-generating sequence with specificity for gene-of-origin, or family-of-origin, sequences. This would occur if an advantage is realized when the target gene or genes are brought under negative regulation by the miRNA, and would lead to selection of the miRNA sequence. The miR824-*AGL16* regulatory pair may exemplify this evolutionary track. The third fate is chance acquisition of targeting specificity for a novel target gene or family, followed by stabilization through selection in the event of an advantage. miR856, which may target transcripts from both gene-of-origin (*ZAT1*) and a novel gene (*CHX18*), could conceivably fall into this category, although it should be noted that there are no direct data supporting a functional role for miR856. We postulate that many of the conserved *MIRNA* genes arose through the latter two routes, and have lost sequence relatedness to their genetic origin loci due to drift of foldback arm sequences outside of the miRNA/miRNA-complementary regions (Figure 4).

While this paper was under review, an article from Rajagopalan et al. [47] describing results from deep sequencing of *Arabidopsis* small RNAs (887,000 reads) was published. They identified and named 32 new miRNAs (miR822-miR853), and another 39 candidate miRNAs that were not named. Sixteen of the named miRNAs, and eight of the candidates, were identified as miRNAs in this study (Table 2). In this paper, 16 miRNAs (miR845b, miR856-miR870) were named, eight of which were identified only here (Table 2). Differences in tissue sampling and sequencing depth likely account for most of the differences in miRNAs identified between the two studies.

## Material and Methods

### *Arabidopsis* mutants and microarray samples

Mutant lines for *dcl1-7*, *dcl2-1*, *dcl3-1*, *dcl4-2*, *rdr1-1*, *rdr2-1*, *rdr6-15* and *hen1-1* were described previously [34], [36]–[38], [48], [49]. All mutants were in the Col-0 background except for *hen1-1* which is in the La-*er* background. All microarray data were generated using Affymetrix ATH1 arrays and are available at Gene Expression Omnibus (GEO) ([50], [51]; http://www.ncbi.nlm.nih.gov/geo/). Col-0 (control for *dcl1-7*) and *dcl1-7* data were from experiments described in Xie et al. [38] (GEO accession GSE3011, samples GSM65938, GSM65939, GSM65940, GSM65941, GSM65942 and GSM65943), and La-*er* (control for *hen1-1*) and *hen1-1* data were from Allen et al. [34] (GEO accession GSE2473, samples GSM47014, GSM47015, GSM47016, GSM47034, GSM47035 and GSM47036).

### Small RNA libraries and ASRP database

Small RNA libraries and database construction were described by Kasschau et al. [28]. Briefly, small RNA analysis for wild-type (Col-0), *dcl1-7*, *dcl2-1*, *dcl3-1*, *dcl4-2*, *rdr1-1*, *rdr2-1*, and *rdr6-15* inflorescence tissue was done by picoliter-scale pyrosequencing (454 Life Sciences [30]). Small RNA preparations from Col-0 and *rdr6-15* whole seedling, and leaf samples of Col-0 that were either uninoculated or inoculated by *P. syringae* pv. tomato (DC3000*hrcC*) for 1 hr and 3 hr, were also analyzed. Methods for normalization of reads were described previously [28]. All small RNA sequences are available for download GEO accession GSE6682 and the ASRP Database (http://asrp.cgrb.oregonstate.edu/db/).

### miRNA identification and target prediction

A set of computational filters based on those developed by Xie et al. [31] were used to identify new miRNAs from among sequences in the ASRP database (http://asrp.cgrb.oregonstate.edu/db/), but with the following modifications. First, only small RNAs that were represented by two or more reads were considered. Second, small RNAs arising from repeat elements [32] and bidirectional siRNA clusters [28] were removed. Third, computational assessment of foldback structure was done with sequences containing 250 nts on each side of candidate miRNAs using RNAfold, Vienna RNA package, version 1.6.1 [52].

miRNA targets were computationally predicted as described [34]. Briefly, potential targets from FASTA searches (+15/−10 match/mismatch scoring ratio, -16 gap penalty and a RNA scoring matrix) were scored using a position-dependent, mispair penalty system [34]. Penalties were assessed for mismatches, bulges, and gaps (+1 per position) and G∶U pairs (+0.5 per position). Penalties were doubled if the mismatch, bulge, gap, or G∶U pair occurred at positions 2 to 13 relative to the 5′ end of the miRNA. Only one single-nt bulge or single-nt gap was allowed. Based on a reference set of validated miRNA targets, only predicted targets with scores of four or less were considered reasonable. Conservation between *Arabidopsis* and Poplar (*P. trichocarpa*) was assessed by FASTA search, foldback analysis, and detection of similar target sequences [18], [48].

### miRNA target validation assays

Target validation using a 5′ RACE assay was done with the GeneRacer Kit (Invitrogen, CA) as described previously [22], [34], [53], [54]. Poly(A)+ mRNA was isolated from seedling (7 day) and inflorescence tissue (28 day, stage 1–12 flowers) of Col-0 plants, ligated to adaptor, converted to cDNA and subjected to two rounds of PCR amplification using gene-specific and adaptor-specific primers [22], [34], [53], [54]. Amplified products were gel-purified, cloned and sequenced. Gene-specific primer sequences for miRNA targets that were successfully validated are shown in Table S4.

### Foldback sequence similarity analysis

The sequences comprising foldbacks from all *MIRNA* loci (Tables 1 and 2) were identified using RNAfold. Foldback sequences were subjected to FASTA searches against the *Arabidopsis* gene and transcript databases [22]. The 5′ and 3′ arms of foldbacks that had gene hits with E-values lower than 0.05 were individually aligned to the top four FASTA hits using NEEDLE [55]. Each arm was also randomly shuffled 1,000 times using SHUFFLESEQ [55] and realigned to each of the top four FASTA hits. The mean ±standard deviation of the randomized sequence scores was calculated. A Z-score was calculated for each arm-gene pair by subtracting the average score of the randomized sequence alignments from the score of the arm-gene alignment and then dividing by the standard deviation of the randomized alignments. This was repeated for arms in which the miRNA or miRNA-complementary sequences were deleted from their respective arms. The deleted arms were aligned with gene sequences in which the target sequence was correspondingly deleted. Intact foldback arms and most-related gene sequences were also aligned and viewed using heat maps with T-COFFEE [56].

## Supporting Information

Table S1 Additional predicted targets for eight *MIRNAs*
(0.02 MB XLS)

Table S2 Summary of *MIRNA* foldback sequences.
(0.02 MB XLS)

Table S3 *MIRNA* loci with sequence similarity to protein-coding genes.
(0.02 MB XLS)

Table S4 Gene-specific PCR primers for successful validation of miRNA targets by 5′ RACE.
(0.02 MB XLS)
